# Formulation, optimization, and nephrotoxicity evaluation of an antifungal in situ nasal gel loaded with voriconazole‒clove oil transferosomal nanoparticles

**DOI:** 10.1080/10717544.2021.1992040

**Published:** 2021-10-20

**Authors:** Ahmed K. Kammoun, Alaa Khedr, Maha A. Hegazy, Ahmad J. Almalki, Khaled M. Hosny, Walaa A. Abualsunun, Samar S. A. Murshid, Rana B. Bakhaidar

**Affiliations:** a Department of Pharmaceutical Chemistry, Faculty of Pharmacy, King Abdulaziz University, Jeddah, Saudi Arabia; b Analytical Chemistry Department, Faculty of Pharmacy, Cairo University, Cairo, Egypt; c Department of Pharmaceutics, Faculty of Pharmacy, King Abdulaziz University, Jeddah, Saudi Arabia; d Department of pharmaceutics and industrial pharmacy, Beni Suef University, Ben-Suef, Egypt; e Department of Natural products and Alternative Medicine, Faculty of Pharmacy, King Abdulaziz University, Jeddah, Saudi Arabia

**Keywords:** Antifungal, clove oil, box‒behnken design, voriconazole, transferosomes, rhinosinusitis

## Abstract

Fungal infections of the paranasal cavity are among the most widely spread illnesses nowadays. The aim of the current study was to estimate the effectiveness of an in situ gel loaded with voriconazole‒clove oil nano-transferosomes (VRC-CO-NT) in enhancing the activity of voriconazole against *Aspergillus flavus*, which causes rhinosinusitis. The nephrotoxic side effects of voriconazole may be reduced through the incorporation of the clove oil, which has antioxidant activity that protects tissue. The Box‒Behnken design was applied to formulate the VRC-CO-NT. The particle size, entrapment efficiency, antifungal inhibition zone, and serum creatinine concentration were considered dependent variables, and the soybean lecithin, VRC, and CO concentrations were considered independent ones. The final optimized formulation was loaded into a deacetylated gellan gum base and evaluated for its gelation, rheological properties, drug release profile, permeation capabilities, and in vivo nephrotoxicity. The optimum formulation was determined to be composed of 50 mg/mL lecithin, 18 mg/mL VRC, and 75 mg/mL CO, with a minimum particle size of 102.96 nm, an entrapment efficiency of 71.70%, an inhibition zone of 21.76 mm, and a serum creatinine level of 0.119 mmol/L. The optimized loaded in situ gel released 82.5% VRC after 12 hours and resulted in a 5.4-fold increase in drug permeation. The in vivo results obtained using rabbits resulted in a nonsignificant differentiation among the renal function parameters compared with the negative control group. In conclusion, nasal in situ gel loaded with VRC-CO-NT is considered an efficient novel carrier with enhanced antifungal properties with no signs of nephrotoxicity.

## 1. Introduction

Overall Fungal rhinosinusitis is a condition that includes several processes, which differ in biological importance, clinical significance, and histopathological manifestations. It is caused by fungi such as *Aspergillus* and the zygomycetous and dematiaceous species (Montone, 2016). The disease may be acute, subacute, or chronic (Chakrabarti et al., 2009). It is usually differentiated into invasive and noninvasive types based on whether fungi have invaded the infected tissue or not (Montone, 2014).

Voriconazole (VRC) is a potent second-generation, broad-spectrum antifungal agent. It is a triazole derivative of the parent drug fluconazole (Scott & Simpson, 2007). VRC exerts its effect through inhibition of the cytochrome P450 (CYP)‒dependent enzyme sterol 14-alpha-demethylase. Therefore, it can disrupt ergosterol production, which is necessary for fungal cell membrane biosynthesis, and can lead eventually to fungal cell lysis (Herbrecht, 2004). VRC is well recommended for the management of invasive aspergillosis. It is also widely prescribed for the treatment of some rare fungal infections, such as those caused by *Fusarium* or *Scedosporium* spp., in addition to other fungal infections that are resistant to standard antifungal drugs (Maertens et al., 2016).

The oral forms of VRC are known to produce several side effects, such as hepatotoxicity, visual disturbances, arrhythmias, and severe dermal reactions, in addition to nephrotoxicity (Chris Rathbun & Hoffman, 2002; Miranda-Cadena et al., 2018). However, the conventional local forms of VRC, including sprays, lotions, gels, and creams, are associated with major limitations of dosing accuracy, a short residence time at the site of application, and variations in performance (Jeu et al., 2003).

Therefore, novel formulations that can be delivered and retained at the desired site with the release of a drug for a selected period would be a possible alternative to conventional forms of delivery. Various researchers have used multiple approaches to deliver VRC effectively for improved efficacy, including a mucoadhesive platform, mucoadhesive nano-particles (Rençber & Karavana, 2018), hydrogels (Raju Y et al., 2017), and nano-sponges (Srinivas & Sreeja, 2013).

Eugenol, which is the major constituent of clove oil (CO), is a phenolic compound accounting for 45% to 90% of the oil (Zhang et al., 2009). Several reports have identified the various pleiotropic activities of eugenol, including anticancer (Dervis et al., 2017), antiinflammatory (Kim et al., 2003), antifungal (Darvishi et al., 2013), antibacterial (Hamed et al., 2012), and analgesic effects (Baldisserotto et al., 2018). Its fungicidal effect might be attributed to its interaction with the cell membranes of different fungi, which results in the leakage of its essential elements and ends with cell death (Lee et al., 2007). Several investigations have mentioned the penetration-enhancing action of eugenol (Mutalik & Udupa, 2003). Based on this information, CO could be considered a good candidate for supporting the antifungal effect of the candidate drug in the treatment of fungal infections such as rhinosinusitis.

Intranasal delivery can be an acceptable alternative route for the administration of several drugs (Jogani et al., 2008). The systemic route of agents that belong to the triazole class, such as VRC, is the commonest way of handling *Aspergillus* infections. However, the adverse effects of oral VRC have been well reported and are thought to be a result of pharmacological effects on host tissues (Laverdiere et al., 2014). In addition, oral VRC can inhibit hepatic P450 enzymes and thus interact with several drugs, and this requires very close therapeutic monitoring (Bellmann & Smuszkiewicz, 2017). Therefore, there is an urgent need for other routes that can be substituted for the oral delivery of VRC to avoid such limitations. The intranasal route can be a suitable substitute for the oral delivery of VRC because it limits the drawbacks of the oral route and can alter the treatment risk/benefit ratio favorably (Patra et al., 2018).

Nano-vesicles have had promising outcomes in the nasal delivery of several agents, such as olanzapine, nefopam hydrochloride, and rivastigmine (Abou-Taleb et al., 2018). Nano-transferosomes (NTs) are ultradeformable vesicles made of phospholipids and an edge activator (Hosny et al., 2020). They are known for their high level of elasticity compared with conventional liposomes; this feature might be due to the presence of the edge activator, which is usually a single-chain surfactant that can increase the fluidity of the vesicles’ bilayer (Cevc & Blume, 2003). Several strengths of NTs have been mentioned in the literature, including their (1) delivery of drugs efficiently to target sites; (2) attainment of prolonged drug release; (3) low level of toxicity; and (4) enhancement of drug bioavailability (Gupta et al., 2012; Maheshwari et al., 2012).

In situ gels are gel bases that are applied as liquid drops and change into gel form at the site of application. Their gelation can be triggered by a change in pH or temperature, exchange of solvents, exposure to ultraviolet radiation, or ionic interaction (Hosny et al., 2020). Deacetylated gellan gum (DGG) is an extracellular polysaccharide obtained from the Gram-negative pathogen *Sphingomonas elodea* (Zhu et al., 2015). It has a parallel double-helix structure, and when it is made into a solution with a known concentration, it attains the property of cation-induced gelation (Singh & Lee, 2014). The unique character of DGG has allowed it to be used for oral (Hamed et al., 2012; Darvishi et al., 2013), ocular (Mutalik & Udupa, 2003; Lee et al., 2007; Baldisserotto et al., 2018), and nasal delivery systems (Cao et al., 2009; Galgatte et al., 2014)

Response surface methodology is a widely credited approach in the formulation and development of many drug delivery systems (Hosny et al., 2021). In this methodology, different experimental and polynomial designs are implemented to correlate and map the relations of different experimental domains. For the present study, the BBD was chosen because of its salient features, such as rotatable experimental designs for therapy combinations, the need for fewer experimental runs, its independent nature, and its cost-effectiveness in permeation enhancement and preparation of the formulation (Ahmed et al., 2015).

The current work is designed to formulate an in situ gel base with the help of gellan gum loaded with VRC NTs to enhance VRC’s intranasal permeation for the treatment of rhinosinusitis and minimize the drug’s dose-dependent toxicities.

## 2. Materials and methods

### 2.1. Materials

VRC, DGG and soybean lecithin were procured from Sigma Aldrich (St, Louis, MO, USA). CO was purchased from Avanti Polar Lipids, Inc. (Birmingham, AL, USA). Tween 80 was generously gifted by the Saudi Drugs and Medical Instruments Company, Jeddah, Saudi Arabia. Solvents of a grade suitable for high-performance liquid chromatography were collected from the Merck Group (Darmstadt, Germany). All other reagents and chemicals used were of analytical grade.

### 2.2. Methodology

#### 2.2.1. Experimental design

The Box–Behnken design (BBD) was applied using Design-Expert 12 (Stat-Ease, Inc., Minneapolis, MN, USA) in order to study the interaction effects. Nineteen experimental trials were generated. Table 1 shows the full experimental plan of the coded and actual values of selected variables and the constraints of dependent variables. The selected independent variables were the concentrations of soybean lecithin (mg/mL) (A), VRC (mg/mL) (B), and CO (mg/mL) (C), and the factorial levels for these factors were coded as ‒1 (low level), 0 (medium level), or +1 (high level) (Alhakamy et al., 2020). The particle size (PS, Y_1_), entrapment efficacy (EE%, Y_2_), inhibition zone (Y_3_), and serum creatinine (Y_4_) were chosen as the responses to be evaluated. The analysis of variance (ANOVA) was utilized to analyze the statistical reasoning of the obtained equations. Various models were used to analyze and correlate the experimental results. The best fitting models (main, interaction, or quadratic) were selected based on the statistical parameters and multiple correlation coefficients (R^2^) (Khajeh, 2009).

**Table 1.** Experimental plan for VRC-NT.

Independent Variables	Levels			Dependent Variables	Constraints
	–1	0	+1		
Soybean Lecithin (mg/mL)- A	50	100	150	Particle Size (nm)	Minimum
Voriconazole (mg/mL)- B	10	20	30	EE (%) Inhibition Zone	Maximum Maximum
Clove oil (mg/mL)-C	25	50	75	Serum creatinine	Minimum

#### 2.2.2. Preparation of VRC-CO-NT formulation

In order to make the VRC-CO-NT, a standard thin-layer evaporation technique was used. The impact of the selected variables on different responses was confirmed by applying three factors and three levels of the BBD. As per the anticipated experimental plan (see Table 1), 19 formulations were planned. In the beginning, a round-bottomed flask, which contained a solvent mixture of methanol and chloroform at 2:1 v/v, was utilized. After that, known quantities of soybean lecithin, VRC, Tween 80, and CO were dissolved in a solvent mixture and left to evaporate at 45 °C at 60 rpm (Heidolph rotary evaporator ML/G3, Heidolph Instruments, Schwabach, Germany) until the flask’s inner layers were lined with the formed lipid layer. The excess solvent was removed by vacuum. Subsequently, a thin layer was formed, and it was reconditioned with phosphate buffered saline (PBS) that had a pH of 7.4 and left for 60 minutes at room temperature. The formed vesicles were left to swell at room temperature for 2–3 hours (Qushawy et al., 2018; Hosny et al., 2020). Finally, the formed vesicles were subjected to size reduction through sonication for 15–20 minutes and stored at 4 °C.

#### 2.2.3. Characterization of VRC-NT

##### Vesicle size and zeta potential

A light-scattering method was used to analyze the vesicle size and zeta potential of the prepared VRC-NT using a Malvern Zeta sizer (Malvern instruments, Malvern, UK) (Guo et al., 2000). One mL samples were diluted with double distilled water (1:10 dilution factor) prior to measurement (El Maghraby et al., 2000). The prepared formulations were measured three times (*n* = 3) in order to achieve the averages.

##### Entrapment efficiency percentage

The EE% was measured employing an indirect method. In the beginning, the prepared formulations were poured into a petri dish. Subsequently, the prepared sample was freeze-dried. After that, an amount of acetonitrile was added and mixed vigorously with the dried specimen (Hosny et al., 2020). This dispersion was centrifuged at a speed of 10,000 rpm for 1 hour. The supernatant liquid that formed was removed and rewashed with acetonitrile. After that, all of the contents were dried in a water bath. Finally, a dried extract was obtained by the addition of a small quantity of methanol. It was diluted and measured at an absorbance of 261 nm (El-Emam et al., 2020). The following formula was used to calculate the EE%:

(1) $$EE(\%)=\frac{\mathrm{Ctotal}-\mathrm{Cfree}}{\mathrm{Ctotal}}\ldots \ldots \ldots \ldots .$$

where Ctotal = theoretical concentration and Cfree = drug concentration in the supernatant fluid.

##### Antifungal activity

The activity of the VRC-NT formulations against *A. flavus* was evaluated using a modified, previously reported disk diffusion susceptibility method (Mahtab et al., 2016). According to the guidelines of the Clinical and Laboratory Standards Institute, an *A. flavus* suspension was made by 0.5 McFarland turbidity standard (106 CFU/mL) using a hemocytometer. An inoculum of 1 mL taken from a suspension of 104 CFU/mL was applied on the surface of Mueller–Hinton agar plates with sterile loops. Disks of 10 mm in diameter containing VRC-NT were then placed over the inoculated plates with sterile forceps. The prepared dishes were incubated for 24 hours at 37 °C, and the diameter of the fungal growth inhibition zone was assessed for each formulation.

##### Serum creatinine measurements

Nineteen rabbits were obtained from the Beni-Suef clinical laboratory center, Beni-Suef, Egypt. All agreements were made as required by the Animal Ethics Committee of the Beni-Suef clinical center and the Declaration of Helsinki (Approval No. 26-06-2020). Each animal received a VRC-NT formulation with a drug concentration of 3 mg/kg twice a day for ten days. Animals were adapted at 20 ± 1 °C for a total duration of 14 days under normal conditions (12/12-hour dark/light cycle) and allowed free access to water and food.

Blood samples of 300 µL were withdrawn from each animal via margenal ear vien in blood collection vials. Test samples were obtained on the 1^st^, 7^th^, and 14^th^ days (Anwar et al., 2020). Samples were centrifuged at 9000 rpm for 15 minutes. Separated serum was stored at −20 °C for further analysis. The serum creatinine was measured using commercially available kits (*Quimica Clinica Aplicada S.A. kit, Amposta*, Spain).

#### 2.2.4. Optimization of VRC-CO-NT

The Design-Expert program employed the desirability function to specify the optimized formulation. The optimization process was done to select a formulation with the maximum EE% and inhibition zone diameter and the minimum PS and serum creatinine level. The formulation chosen was developed and tested and finally compared with the expected values of studied responses. Eventually, the optimum formulation was incorporated into an in situ gel base.

#### 2.2.5. Ex vivo *permeation studies*

First, fresh nasal mucosa was collected carefully from goat nasal cavities supplied by a local slaughterhouse. The provided tissues were placed separately on a Franz diffusion cell that was filled with 7 mL PBS (pH = 6) in a receptor chamber. A 20-minute preincubation time was provided for this solution (Buss et al., 2001). After that, an amount of the optimized drug formulation (1 g) and 1 mL of the pure drug suspension were kept in the donor chamber that exhibited an effective permeation area of 5 cm^2^. Experiment was carried out at at a temperature of 37 ± 0.5 °C. Subsequently, several samples (0.5 mL) were withdrawn at regular time intervals, and a high-performance liquid chromatography method was adopted to analyze the withdrawn samples with a column: Phenomenex luna C18 (250 × 4.6 mm, 5 μm); mobile phase: 55/45; organic phase (41/18/10 methanol/acetonitrile/tetrahydrofuran)–buffer (2.5 mmol l^−1^ EDTA-2Na); flow rate: 1.0 mL min^−1^; column temperature: 30 °C; detection wavelengths: 383 and 303 nm; injection volume: 20 μL. Different permeation parameters, such as the cumulative amount permeated per unit area (Q_24_, µg/cm^2^), diffusion coefficient (D), enhancement ratio (ER), permeability coefficient (P), and steady-state flux (Jss), were calculated from the data obtained.

(2) $$\mathrm{Jss}=\frac{Qt}{A}\;.t$$

(3) $$P=\frac{\mathrm{Jss}}{C0}$$

(4) $$ER-\frac{\;Q24\;of\;\mathrm{studied}\;\mathrm{formulations}}{Q24\;of\;\mathrm{pure}\;\mathrm{drug}\;}$$

where,Q_t_ is the quantity of drug passed through the mucosa into the receptor champer (µg), A is the effective diffusion surface area (cm^2^), and t is the time of exposure (min), Jss is the flux calculated at the steady state and C_0_ is the initial drug concentration in the donor compartment.

#### 2.2.6. In situ gel preparation

DGG was sprayed into 6 mL distilled water at a high temperature (80 ± 2 °C). Then, the formed solution was stirred steadily until the polymer was dissolved. The formed dispersion was left to cool for a whole night (Ahmed et al., 2015; Hosny et al., 2021). After that, 4 mL of the optimized VRC-NT formulation was added to the formed solution and mixed thoroughly until a homogenous mixture was obtained.

#### 2.2.7. Evaluation of VRC-NT-loaded in situ gels

##### Critical ionic concentration

Ionic activation leads to phase transformation, which is critically important for in situ gels. First, different amounts of nasal fluids and DGG (1 mL of 0.5% solution) were mixed. After that, the formed solution was poured into glass bottles and kept in a water bath at 32 °C. The filled bottles were turned over after 20 seconds. The bottle’s contents adhered to the bottom instead of flowing downward when the gels form. This was noted as +. The least quantity of nasal fluid required to create a gel was termed the critical ionic concentration (CIC) (Alhakamy et al., 2020).

##### Expansion coefficient

When the solution is transformed into a gel, its volume may increase. Discomfort in the nasal region may occur due to this volume expansion.

The expansion coefficient (S%) was assessed by mixing 1 mL of DGG solution (0.5%) with 0.25 mL of nasal fluid in a graduated test tube. Then, it was placed on a water bath at 32 °C (VI = 1.25 mL). After that, 2 mL of the optimized preparation was added to the above solution (VT = 3.25). The final volume of the prepared sample (VG) was obtained by measuring a cylinder and recorded, and changes in the volume of the samples after the gelation (V_G_) were noted (Khajeh, 2009). The following formula was used to calculate the S%:

(6) (S%) = (VG ‒ VI)/VI × 100…….

##### Rheological properties

A Brookfield Digital Viscometer (AMETEK Brookfield, Middleboro, MA, USA) was utilized to record the apparent viscosity values at 30 seconds before and after the gelation process. The digital viscometer was maintained at 10 rpm.

##### Gel strength measurement

The gel strength is an indication of the viscosity associated with the produced formulation at room temperature. The addition of nasal fluid resulted in the conversion of about 5 g of the formulation into the gel. Afterward, a 3.5-g weight was placed above the obtained in situ gel. The time needed for the weight to reach a depth of 3 mm in the gel is termed the gel strength (Singh et al., 2013).

##### In vitro *drug release studies*

The VRC released from the selected formulation was estimated by dialysis bag. It was filled with 5 mL of the formulation and kept in a vessel holding a PBS of 100 mL (pH = 6); the dissolution medium was kept at 37 ± 1 °C and 50 rpm. Samples were withdrawn at certain intervals (i.e. 1, 2, 4, 6, 8, 10 and 12 h), and the release medium was replenished with an equal volume of fresh medium. Withdrawn samples were analyzed for VRC content at 261 nm.

#### 2.2.8. Nephrotoxicity studies

In this study, 18 rabbits were obtained from the Beni-Suef clinical laboratory center, Beni-Suef, Egypt. All agreements were proclaimed by the Animal Ethics Committee of Beni-Suef clinical center and the Declaration of Helsinki (Approval No. 26-06-2020). The animals were divided into three groups (six animals in each group). The first group was treated with 0.3 mL of intranasal normal saline as a negative control. The second group was treated with intranasal VRC 3 mg/kg dispersed in normal saline as a positive control. The third group was treated with intranasal optimized VRC-CO-NT loaded in in situ gel 3 mg/kg. All rabbits were adapted at 20 ± 1 °C for a total duration of 14 days under normal conditions (12/12-hour dark/light cycle) and allowed free access to water and food. Blood sampling was performed as previously mentioned in the serum creatinine test.

##### Serum biochemical markers

The urea, blood glucose, calcium, sodium, and potassium levels were measured using commercially available kits (*Quimica Clinica Aplicada S.A. kit, Amposta*, Spain).

## 3. Results and discussion

### 3.1. Box–behnken design analysis

**Figure 1.**
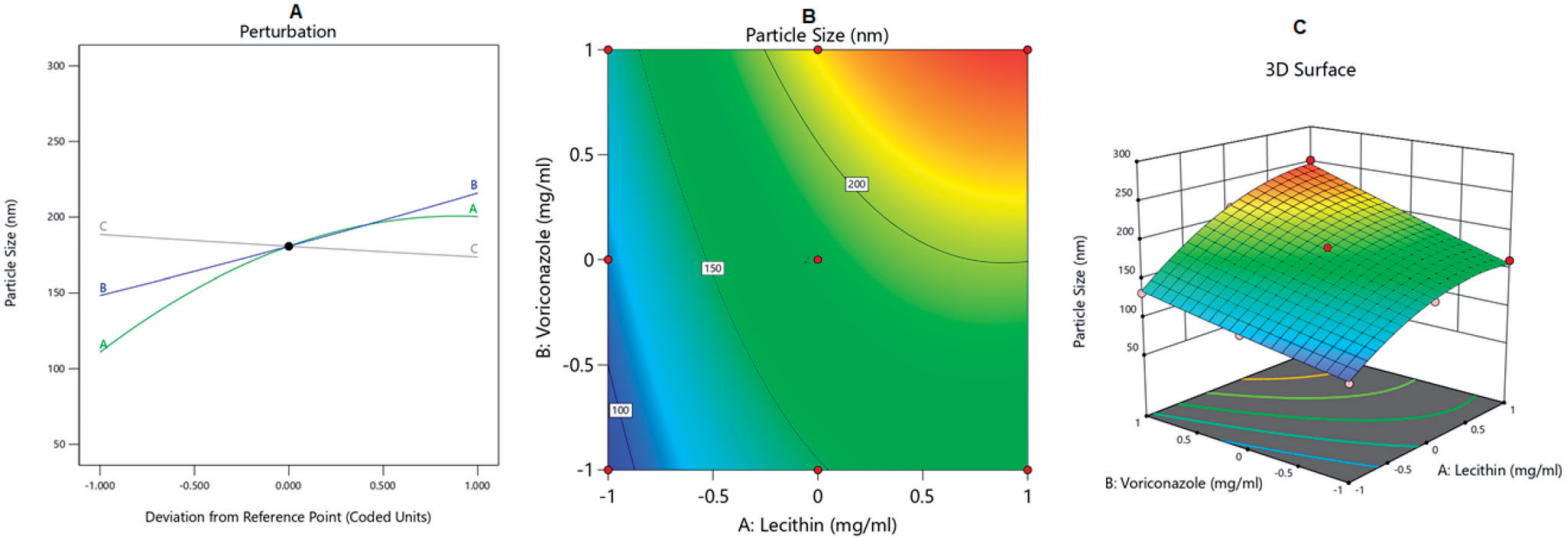
Main effect diagram (A), contour plot (B), and 3D surface plot (C) showing the effects of different independent variables on the particle size of VRC-NT.

**Figure 2.**
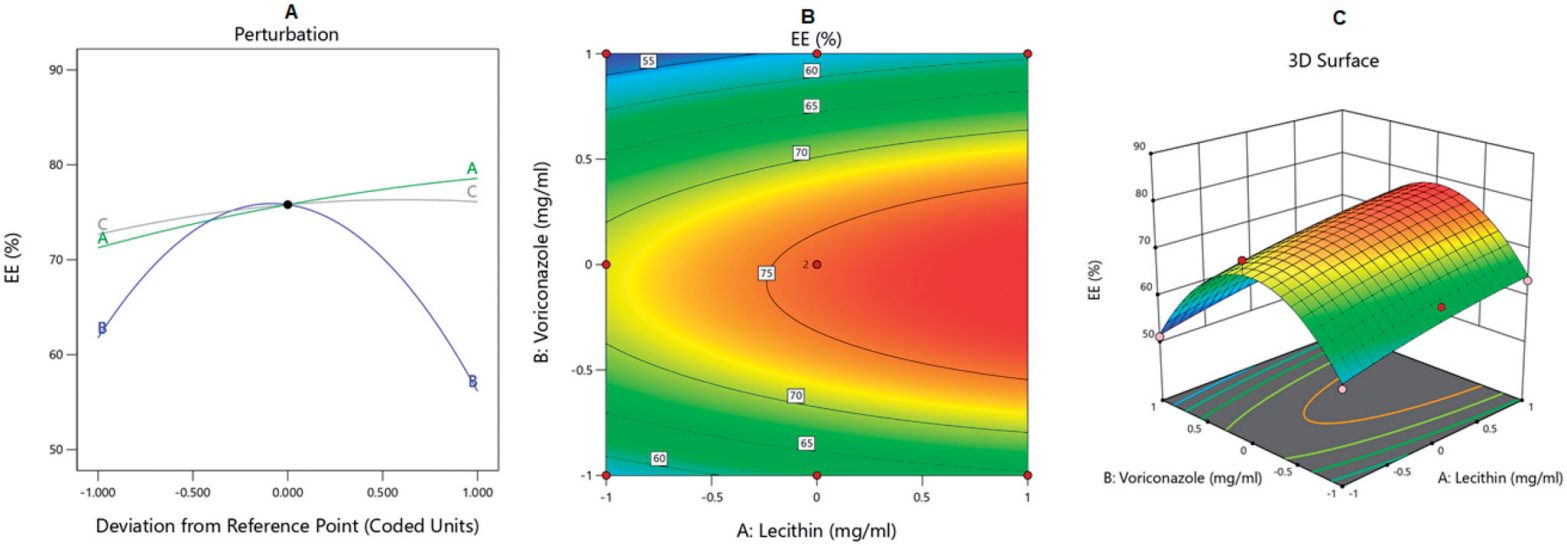
Main effect diagram (A), contour plot (B), and 3D surface plot (C) showing the effects of different independent variables on the EE% of the VRC-NT.

**Figure 3.**
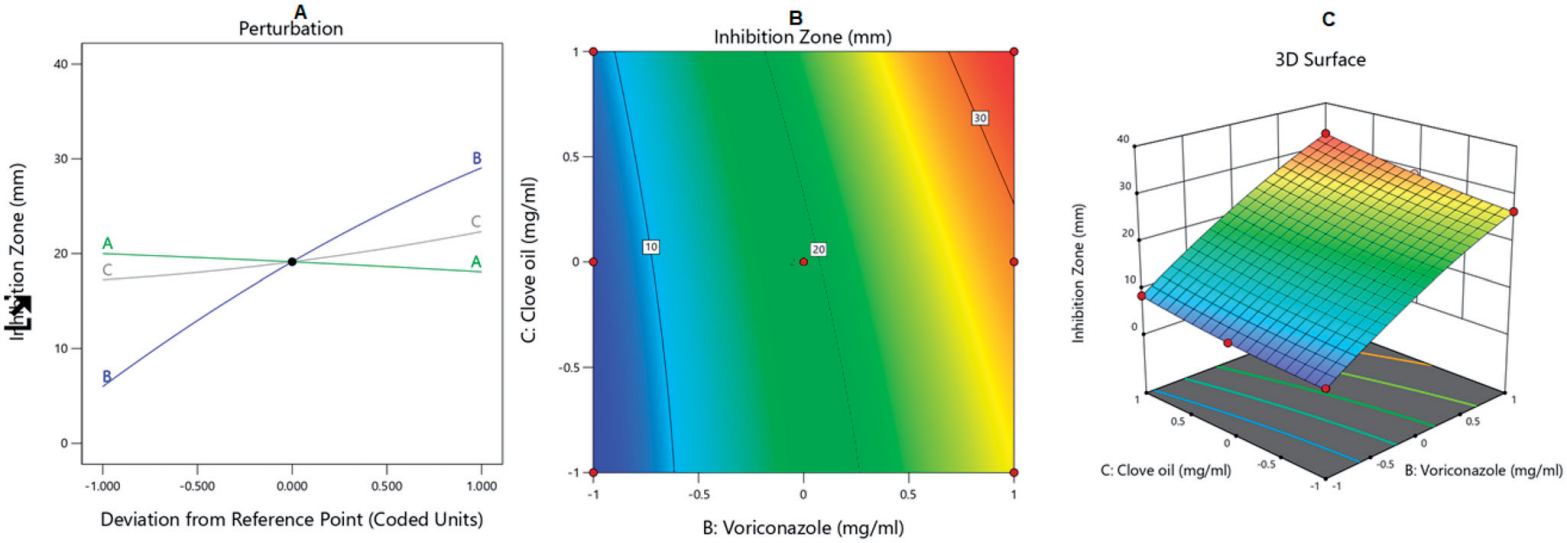
Main effect diagram (A), contour plot (B), and 3D surface plot (C) showing the effects of different independent variables on the inhibition zones of VRC-NT.

**Figure 4.**
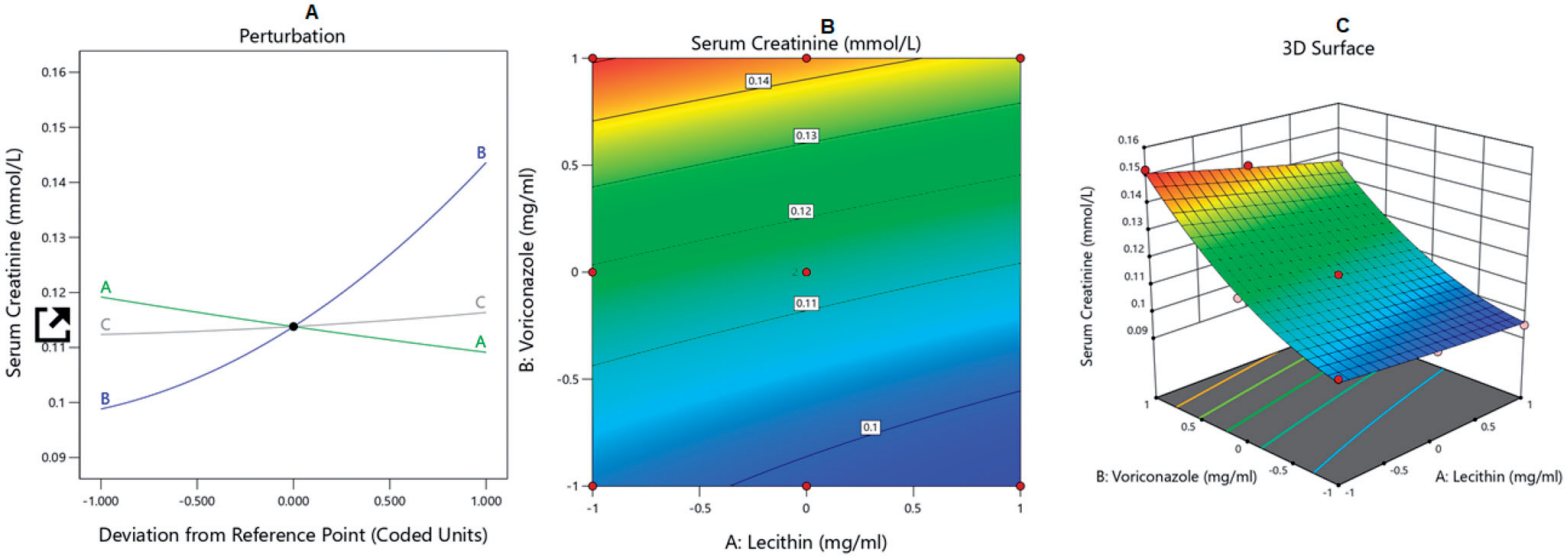
Main effect diagram (A), contour plot (B), and 3D surface plot (C) showing the effects of different independent variables on the serum creatinine levels in animals treated with VRC-NT formulations.

The BBD was used to analyze the optimal levels of the selected factors and their interactions in the measured responses and to test the model adequacy and fit. The analysis of variance and correlation coefficients had a meaning rate of 95% (*p* < .05), and the F measures were adopted to statistically test the validation of the suggested models. A check point analysis was applied to check the accuracy and validity of the suggested statistical models for the forecasting of the measured responses. The response surface plots were drawn. The overlaid region of an overall required response corresponded to the optimal region where the optimum dispersions could be acquired. At the end, the desirability values were assessed and the optimum specifications were compared for the estimation of the formulation. The effect of the independent variables on the dependent responses was explained by three-dimensional (3D) surface, contour, and main effect plots as illustrated in Figures 1–4.

### 3.2. Particle size determinations

Concerning nasal delivery, the nanovesicles’ physicochemical properties are the main parameters in studies of formulation production. The PS of all trial batches was found to be in the range of 87.6–234.6 nm, as shown in Table 2.

**Table 2.** Projected trial formulations and their observed responses as per the Box‒Behnken design.

Run	A: Lecithin	B: Voriconazole	C: Clove oil	Particle size	EE%	Inhibition zone	Serum Creatinine
	mg/mL	mg/mL	mg/mL	nm	%	mm	mmol/L
1	50	10	25	92	53.68	5	0.10165
2	50	10	50	87.6	56.32	7	0.10355
3	50	20	25	120	67.76	18	0.1159
4	50	20	50	110.4	74.26	20.5	0.1178
5	50	20	75	109.2	71.28	23	0.1216
6	50	30	50	132	51.04	30	0.152
7	100	10	25	156	58.96	5	0.0969
8	100	10	50	144.9	64.86	6	0.0988
9	100	10	75	144	61.6	8.5	0.1007
10	100	20	50	184.8	74.8	19	0.114
11	100	20	50	179.4	73.92	19	0.114
12	100	30	25	228	52.8	26.5	0.141
13	100	30	50	212.75	57.34	29	0.1444
14	100	30	75	205.85	55.44	33	0.14725
15	150	10	50	161	63.36	5	0.095
16	150	20	25	204	76.56	16.5	0.1102
17	150	20	75	188.6	78.32	21	0.1121
18	150	30	50	252	57.2	27.5	0.13585
19	150	30	75	234.6	62.04	32	0.1387

Particle size values were analyzed by the quadratic model of polynomial analysis. The BBD design illustrated the efficiency of the suggested model in investigating the significance of the lecithin (A), VRC (B), and CO (C) concentrations on the NT particle size. The obtained statistical model had an adjusted R^2^ of 0.9921; this was in accordance with the predicted R^2^ value of 0.9727, as shown in Table 3. Statistical analysis of the obtained data resulted in the following equation:

(7) $$\mathrm{Particle}\;\mathrm{Size}\;=\;+\;180.73\;+\;44.73A\;+\;33.77B\;‒\;7.47C\;+\;11.24AB\;‒\;1.56AC\;‒\;2.95BC\;‒\;25.08A^{2}\;+\;1.40B^{2}+0.3580C^{2}$$

**Table 3.** Regression analysis results for the Y_1_, Y_2_, Y_3_, and Y_4_ responses.

Dependent variables	R^2^	Adjusted R^2^	Predicted R^2^	p-value	F-value	Adequate precision
Y_1_	0.9961	0.9921	0.9727	0.0001	253.21	49.0846
Y_2_	0.9714	0.9429	0.8995	0.0001	34.02	17.8396
Y_3_	0.9994	0.9989	0.9973	0.0001	1745.04	116.1858
Y_4_	0.9972	0.9944	0.9817	0.0001	356.89	54.3469

As can be seen, factors A and B had a positive effect on the particle size at a *p*-value of less than .0001, whereas factor C had a negative effect at a *p*-value of less than .0006. Increasing the soybean lecithin concentration resulted in larger vesicles; this might be explained by the ability of lecithin’s main constituent, phosphatidylcholine, to impart negative charges to VRC-NT. This allowed more aqueous media to be incorporated within the NT core, resulting in the production of larger vesicles (Singh et al., 2013). Additionally, higher lecithin concentrations could increase the membrane’s rigidity and formulation’s viscosity, making it harder to downsize the developed vesicles.

Increasing the VRC concentration also yielded vesicles with larger diameters. This might be ascribed to the availability of more drug to be accommodated in the NT shell, forming larger vesicles. On the other hand, the decrease in PS observed with the increase in the CO concentration might be because of the decrease in surface free energy that occurs upon an increase in total lipid. Similar findings were reported in the literature (Guo et al., 2000). The effect of the independent variables on the PS of VRC-NT is explained in the 3D surface, contour, and main effect plots in Figure 1.

### 3.3. EE% evaluation

The EE% values of the developed VRC-NT formulations ranged between 51.04% and 78.32%, as seen in Table 2.

A quadratic model of polynomial analysis was followed for the EE% values. The adopted design calculated the efficiency of the proposed model to explore the significant action of the lecithin (A), VRC (B), and CO (C) concentrations on the NT EE%. The suggested model attained an adjusted R^2^ of 0.9429, which was close to the value of the predicted R^2^ of 0.8995, as shown in Table 3. The analysis of variance of the collected data gave the following equation:

(8) $$\text{EE\% }=\;+\;75.81\;+\;3.66A\;‒\;2.81B\;+\;1.72C\;+\;0.1519AB\;‒\;0.0681AC\;+\;0.3719BC\;‒\;0.8858A^{2}\;‒\;16.85B^{2}\;‒\;1.41C^{2}$$

As noted, factors A and C had a positive effect on the EE% at *p*-values of less than .0004 and 0.0340, respectively. The increase in lecithin concentration (A) led to an increase in VRC incorporation into the NT. This could be attributed to the increase in membrane viscosity and rigidity with higher lecithin concentrations, leading to a decrease in drug leakage from the transferosomal membrane (Singh et al., 2013). Additionally, the increase in the CO concentration could have increased the solubility of the VRC in the vesicle’s shell, leading to the incorporation of more drug. Interestingly, factor B and its quadratic term, B^2^, had a significant effect on the EE%. As can be observed in Figure 2(A), this means that the peripheral levels of factor B had a negative effect on the EE% but that the middle level had a positive effect and led to higher EE% values. Such outcomes might be due to leakage and the precipitation of excess VRC from NTs. Consequently, it could be assumed that NTs could increase the VRC solubility to a certain extent, after which VRC is precipitated due to the saturation of the transferosome bilayers. Similar conclusions were reported in the literature (Salem et al., 2021). Effects of the independent variables on the EE% are illustrated by the main effect diagram and contour and 3D surface plots in Figure 2.

### 3.4. Evaluation of VRC-NT antifungal activity

The antifungal activity of the VRC-loaded NTs was evaluated against the *A. flavus* fungus by measuring the inhibition zone’s diameter of fungal growth in plates treated with VRC-NT. As shown in Table 2, the diameter of the inhibition zone of *A. flavus* ranged from 5 to 32 mm. Inhibition zones against *A. flavus* acquired a quadratic model of polynomial analysis based on the adopted design, which examined the effect of the independent variables on the inhibition zones’ diameters.

The predicted R^2^ of the developed model was 0.9989; this was in good agreement with an adjusted R^2^ of 0.9973, as presented in Table 3. Analysis of the collected data using ANOVA resulted in the following equation:

(9) $$\mathrm{Inhibition}\;\mathrm{Zone}\;=\;+\;19.13\;‒\;0.9636A\;+\;11.53B\;+\;2.54C\;‒\;0.1404AB\;‒\;0.1404AC\;+\;0.7346BC\;‒\;0.0970A^{2}\;‒\;1.61B^{2}\;+\;0.6523C^{2}$$

The above equation clarifies that factor (A) exerted a significantly negative effect (*p* < .0001) on the zone of inhibition against *A. flavus,* and this implies that increasing the lecithin concentration will decrease the inhibition zone’s diameter. Such a result can be attributed to the effect of lecithin on the PS of the vesicles because increasing the lecithin concentration led to an increase in the PS values and, consequently, a smaller surface area would be available for the VRC to penetrate the fungal cells to cause lysis (Salem et al., 2020). However, the VRC (B) and CO (C) concentrations had a significant positive effect on that response (*p* < .0001).

The antifungal action of CO against *A. flavus* might be caused by its eugenol constituent, which is well reported to have a good antifungal effect. As was reported in the literature, eugenol can cause potassium leakage from fungal cells and, consequently, prevent the uptake and use of energy by such cells and disrupt the fungal cell membrane (Hosny et al., 2020).

As for the VRC, its antifungal activity is reported to be due to its ability to inhibit the fungal cytochrome P450 (CYP)‒dependent enzyme sterol 14-alpha-demethylase, which is a key element in ergosterol biosynthesis, resulting in fungal cell wall disruption and cell lysis (Scott & Simpson, 2007). Effects of the independent variables on the inhibition zone are illustrated in the main effect diagram and contour and 3D surface plots in Figure 3.

### 3.5. Serum creatinine measurements

Serum creatinine is a specific marker for kidney damage because renal insufficiency is the only parameter that elevates serum creatinine in mammals. The serum creatinine levels observed in animals after exposure to VRC-loaded NTs ranged from 0.095 to 0.14725 mmol/L, as shown in Table 2. Serum creatinine levels acquired a quadratic model of polynomial analysis based on the adopted design, which examined the effect of the independent variables on serum creatinine levels.

The predicted R^2^ of the developed model was 0.9944; this was in good agreement with an adjusted R^2^ of 0.9817, as presented in Table 3. Analysis of the collected data using ANOVA resulted in the following equation:

(10) $$\mathrm{Serum}\;\mathrm{Creatinine}\;=\;+\;0.1138‒\;0.0050A\;+\;0.0224B\;+\;0.0020C\;‒\;0.0018AB\;‒\;0.0009AC\;+\;0.0007BC\;+\;0.0004A^{2}\;+\;0.0074B^{2}+\;0.0006C^{2}$$

It was observed that factor (A) exerted a negative effect on serum creatinine levels in animals (*p* < .0001). The decrease in response Y4 following the increase in lecithin concentration could be explained based on the PS results. The increase in the lecithin concentration resulted in an increase in PS values, which caused smaller surface areas to be available for drug penetration and absorption and, hence, smaller amounts to reach the kidneys.

On the contrary, factors (B) and (C) exerted a significantly positive effect (*p* < .00001) on serum creatinine levels. As expected, the increase in VRC concentration (factor B) led to an increase in serum creatinine levels, because it is well reported that triazoles, the class to which VRC belongs, are known for their nephrotoxic side effects (Chris Rathbun & Hoffman, 2002). Moreover, the increase in CO increased the serum creatinine levels as expected, because it decreased the size of the nano-vesicles and offered a larger surface area for drug absorption and also helped in the solubilization of VRC and, hence, its absorption. Effects of the independent variables on serum creatinine levels are illustrated by the main effect diagram and contour and 3D surface plots in Figure 4.

### 3.6. Optimization of VRC-NT formulations

**Figure 5.**
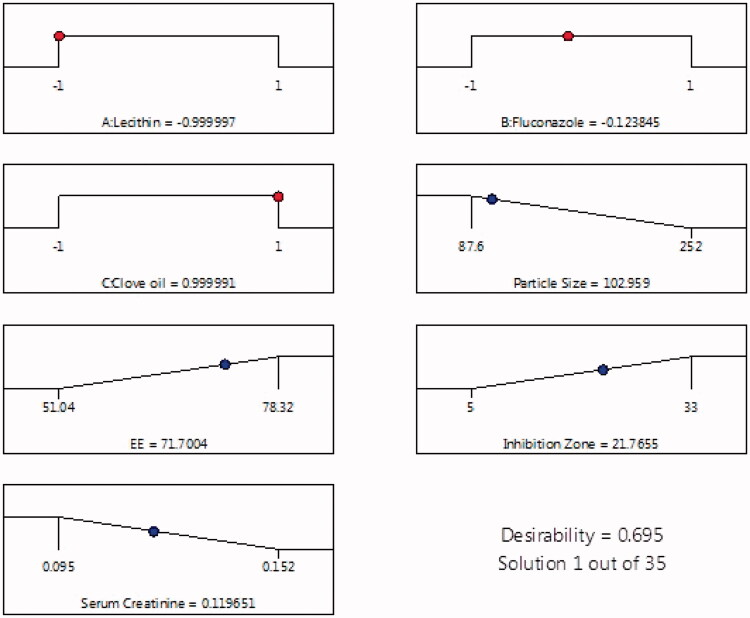
Desirability ramp shows the levels for the independent variables and predicted values for the responses of the optimum formulation.

The global desirability function (D) was used to optimize the sequence of models obtained from statistical research analysis (Alkhalidi et al., 2018; Alghaith et al., 2021). Each response was set to limits (EE and inhibition zone to a maximum, and PS and serum creatinine to a minimum) to frame an overlay graph to optimize the independent variables. The independent variables (optimal concentration) indicated a 0.695 D value (maximum) for responses in the desirability plot. Therefore, implementing these settings can result in attaining a minimum size (102.96) and serum creatinine level (0.119 mmol/L) while obtaining a maximum EE (71.70%) and inhibition zone (21.76 mm) (Figure 5). By using these concentrations, an optimized formulation was prepared, and it validated the experimental design. Table 4 shows that the observed and predicted values of the optimum formulation parameters were in very close agreement and had no major differences (*p* > .05), and this proved the equations’ predictability and validity.

**Table 4.** Composition and actual and predicted responses of the optimal VRC-NT formulation.

Factor	Coded level	Response Variable	Actual value	Predicted value	% Prediction error^a^
A:Soybean lecithin conc.	0.99	Particle size (nm)	103.5	102.96	0.266
B:VRC conc.	–0.12	EE %	72.60	71.70	0.011
C:Clove oil conc.	0.99	Zone of inhibition against Aspergillus flavus (mm)	23	21.76	0.0539
		Serum creatinine levels(mmol/L)	0.117	0.119	−0.017

^a^Calculated as [Actual-predicted/Actual] × 100.

#### Check point analysis

Values of the adjusted and expected R^2^ of the measured responses were in close agreement, suggesting that the developed design had a valid and significant prediction capacity. In addition, the ratios of the experimental values to the predicted values had a percentage error lower than 10%. The residuals observed between the predicted and experimental responses were acceptable, proving the lack of curvature in the responses and the model fitness and adequacy (Table 4).

#### Ex vivo *permeation studies*

Table 5 shows the ex vivo permeation results of the optimized formulation, the optimized formulation without CO, and the drug aqueous suspension through the nasal membrane. All formulations showed significant differences for all the evaluation parameters. The ex vivo permeation findings revealed that 83.4% of the total drug in the optimized VRC-NT formulation permeated the membrane. On the other hand, only 47.3% of the drug from the optimized formulation prepared without CO permeated the membrane. This confirmed that CO enhances permeation through the nasal membrane by altering both the lipid and polar permeation pathways (Hosny et al., 2020). These results could also be ascribed to the components of the transferosomes. The phospholipids and the edge activator used could have reduced the interfacial tension at the surface of the nasal mucosa; this would have been in addition to the reasonable affinity of phospholipids for the biological membrane. Furthermore, the known elasticity and deformability of the transferosomes that are attributed to their EA content could have enhanced the VRC permeation of the nasal mucosa (Khallaf et al., 2020).

**Table 5.** Ex vivo permeation results.

Permeation Parameters	Optimized formulation	Optimized formulation Prepared without clove oil	VRC Aqueous suspension
Cumulative amount permeated (μg/cm^2^)	9813 ± 912	5614 ± 392	1813 ± 152
Cumulative percent permeated	83.4%	47.3%	
Steady state flux, Jss, (μg/cm^2^.min)	5.144 ± 1.11	3.287 ± 0.60	1.254 ± 0.31
Permeability coefficient, P, (cm/min)	2.414 × 10^–3^	1.754 × 10^–3^	0.411 × 10^–3^
Diffusion coefficient, D, (cm^2^/min)	19.37 × 10^–5^	13.22 × 10^–5^	5.66 × 10^–5^
Enhancement factor (EF)	5.41	3.07	–

### 3.7. Evaluation of VRC-NT in situ gels

#### In vitro *drug release studies*

**Figure 6.**
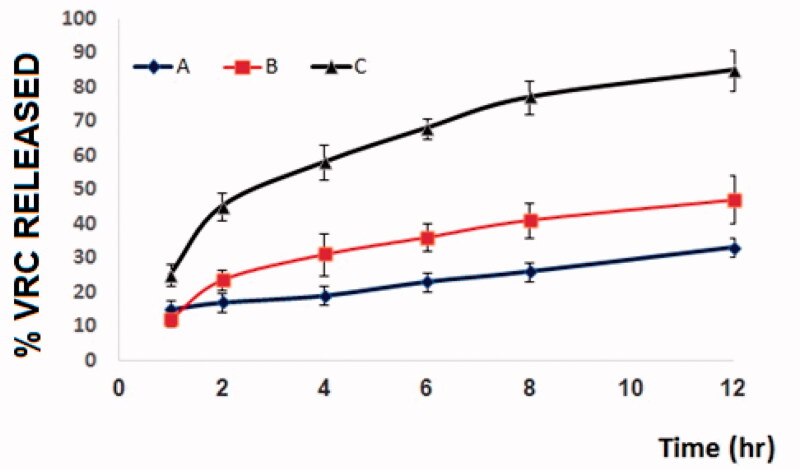
In vitro drug release study for (A) VRC release from drug aqueous suspension, (B) VRC release from pure VRC-loaded in situ gel, and (C) VRC release from optimized VRC-NT-loaded in situ gel formulation.

VRC release from the optimized formulation was determined using a dialysis bag method, and the results are shown in Figure 6. Studies were performed for a 12-hour period of time. A low percentage of drug release was observed from the drug aqueous suspension and the VRC-loaded in situ gel until the end of the experiment. This could be due to a poor solubility of VRC from such formulations. VRC was released from the optimized formulation in an amount of 82.25%. Enhanced drug release was observed from NTs, and this could be credited to the reduced particle size. An initial burst release was observed due to incomplete gel formation followed by gelation, which causes slow drug release to reach a steady-state concentration.

#### Nephrotoxicity studies

The kidney function parameters were measured in rabbit plasma after administration of the VRC aqueous suspension and VRC optimized nano-transferosomal in situ gel. They were measured in the control group, as well.

There was an obvious reduction in the renal parameters in animal groups treated with either the drug suspension or the VRC-NT-loaded in situ gel compared with the control group. VRC contributed to a reduction in the glucose level in the test groups compared with the control group because VRC could have caused renal tubular damage and diminished the glucose tubular reabsorption process (Chris Rathbun & Hoffman, 2002). The VRC also contributed to the elevation of serum creatinine and urea levels. This could be ascribed to renal tubule damage and its negative effect on the glomerular filtration process of urea, leading to the accumulation of such parameters in plasma (Hosny & Alhakamy, 2020). Furthermore, VRC apparently caused hyperkalemia in treated animals; such an effect could be due to the ability of VRC itself to cause hypoaldosteronism. Similar findings were reported in the literature (Somani et al., 2000). Hypoproteinemia might be responsible for the decreased serum calcium levels in the test samples. The sodium levels were slightly varied.

**Figure 7.**
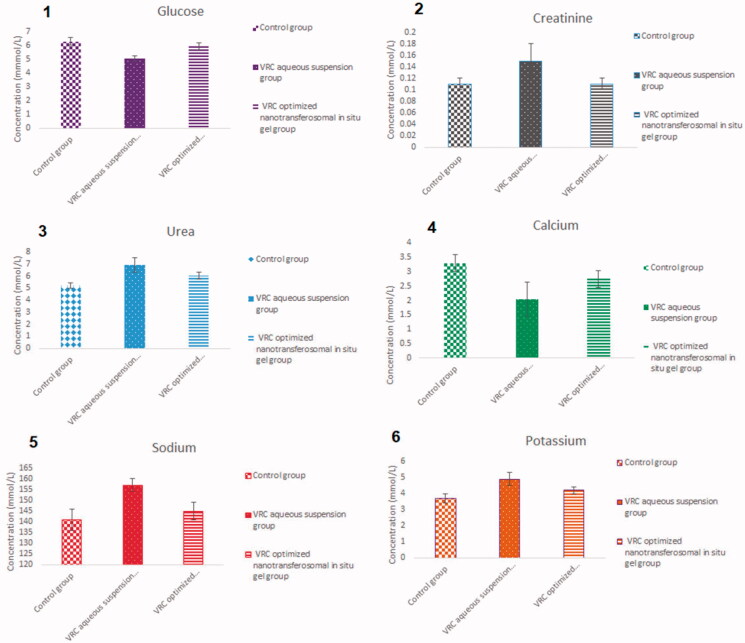
Kidney function parameters: (1) glucose, (2) creatinine, (3) urea, (4) calcium, (5) sodium, and (6) potassium (mmol/L), measured in rabbit plasma after 14 days of treatment.

As can be seen in Figure 7, the renal parameters of the third group, which was treated with VRC-NT-loaded in situ gel, were better than those of the second group, which was treated with the drug aqueous dispersion. Such an observation could lead to the assumption that loading VRC into NTs could have decreased its renal damaging effect by obtaining the optimized formulation with the lowest nephrotoxic effect. Different renal parameters measured in tested animal groups are shown in Figure 7.

This investigation might be limited by the lack of human and clinical studies, therefor, it is strongly recommended to perform future clinical studies to explore the effects of the produced formulation on VRC nephrotoxic side effects.

## 4. Conclusion

The BBD was used to optimize the preparation of VRC-CO-NT in the current research. Individual responses were evaluated for all observable effects, and the influence of variables was statistically evaluated using ANOVA. On the basis of the desirability approach formulation containing 0.99 coded levels of lecithin (mg/mL), ‒0.12 coded levels of VRC (µg/mL), and 0.99 coded levels of CO (µg/mL), the prerequisite of the optimum formulation was achieved. Implementing these results can result in attaining a minimum size (102.96) and maximum EE (71.70%), inhibition zone (21.76 mm), and serum creatinine level (0.119 mmol/L). Optimized NTs were loaded into an in situ gel system to make it more suitable for nasal delivery. The prepared in situ gel had enhanced drug release and permeation. This can be credited to the CO in the formulation. Nephrotoxic studies had results as expected. In conclusion, we can say that the formulated in situ gel was found to be proficient for the nasal delivery of VRC.
